# Factoring semi-primes with (quantum) SAT-solvers

**DOI:** 10.1038/s41598-022-11687-7

**Published:** 2022-05-14

**Authors:** Michele Mosca, Sebastian R. Verschoor

**Affiliations:** 1 Institute for Quantum Computing, University of Waterloo, Waterloo, Canada; 2 Department of Combinatorics and Optimization, University of Waterloo, Waterloo, Canada; 3 David R. Cheriton School of Computer Science, University of Waterloo, Waterloo, Canada; 4 Perimeter Institute for Theoretical Physics, Waterloo, Canada; 5 Canadian Institute for Advanced Research, Toronto, Canada; 6 evolutionQ Inc., Waterloo, Canada

**Keywords:** Computer science, Information theory and computation

## Abstract

The computational difficulty of factoring large integers forms the basis of security for RSA public-key cryptography. The best-known factoring algorithms for classical computers run in sub-exponential time. The integer factorization problem can be reduced to the Boolean Satisfiability problem (SAT). While this reduction has proved to be useful for studying SAT solvers, large integers have not been factored via such a reduction. Shor’s quantum factoring algorithm factors integers in expected polynomial time. Large-scale fault-tolerant quantum computers capable of implementing Shor’s algorithm are not yet available, preventing relevant benchmarking experiments. Recently, several authors have attempted quantum factorizations via reductions to SAT or similar NP-hard problems. While this approach may shed light on algorithmic approaches for quantum solutions to NP-hard problems, in this paper we study and question its practicality. We find no evidence that this is a viable path toward factoring large numbers, even for scalable fault-tolerant quantum computers, as well as for various quantum annealing or other special purpose quantum hardware.

## Introduction

In this work we focus on the problem of factoring semi-primes with SAT-solvers. A semi-prime *N* is a composite of two primes *p* and *q* which are roughly of equal size. These particular composites are conjectured to be hard to factor, in the sense that no (classical) algorithm or heuristic is known to factor semi-primes using only polynomially many resources. This problem has great relevance for the RSA cryptosystem^1^, a widely-deployed public-key cryptosystem. The RSA cryptosystem is founded upon the difficulty of factoring integers: the existence of an efficient factoring algorithm would completely break its security.

Some authors have proposed an alternative approach they refer to as quantum factoring. In this paper, we explain why these approaches, while potentially helpful for studying quantum SAT-solving, are not likely a viable approach to integer factorization and, very importantly, are not a meaningful benchmark for people interested in quantum cryptanalysis of cryptosystems based on the integer factorization problem.

We attempt to generously extrapolate the kinds of speed-ups one might expect for a range of quantum solvers, and find no evidence that this is a viable path toward factoring large numbers, even for scalable fault-tolerant quantum computers, as well as for various quantum annealing or other special purpose quantum hardware.

Some researchers only implement quantum factoring for the purposes of benchmarking the experimental apparatus. There are several more relevant algorithms to implement for the purposes of benchmarking, such as work on randomized benchmarking^2^ or implementations of quantum error correction. Framing the experiments as implementations of quantum factoring can easily be misinterpreted as a meaningful benchmark toward large-scale integer factorization, and we explain in this article why they are not.

For many years cryptographers have tracked and benchmarked progress in classical factorization and attempted extrapolations with an interest in estimating when RSA schemes with moduli of a given length may be broken using the number field sieve^3,4^. The extrapolations take into account estimates of computing power increase and algorithmic improvements.

This paper highlights why none of the current works in the literature on experimental implementations of quantum factoring serve the same purpose. In the absence of a breakthrough that demonstrates factoring can be meaningfully sped up without a fault-tolerant quantum computer, this sort of tracking of the size of numbers quantumly factored will only be meaningful after the implementation of several logical qubits.

One caveat and challenge with tracking and extrapolating is that once fault-tolerant quantum computers start factoring small numbers, a constant factor increase in available quantum resources brings a constant *factor* increase in the size of the number that can be factored (i.e. we go from being able to factor *n*-bit numbers to being able to factor (*cn*)-bit numbers for some $c>1$ that depends on the factor of increase in time and memory) because Shor’s algorithm runs in expected polynomial time. On the other hand, a constant factor increase in classical computing resources only implies being able to factor numbers that are a few bits larger using the number field sieve (i.e. we go from being able to factor *n*-bit numbers to being able to factor $(n + o(n^{2/3}))$-bit numbers). Given these quantum scalings, it will be much harder to reliably extrapolate the size of numbers that can be quantumly factored, and a relatively small change in computing resources or a relatively small algorithmic improvement can have a significant impact on the size of the number that can be quantumly factored. This is one reason why it is valuable to have post-quantum cryptography ready for wide-scale deployment before fault-tolerant quantum computers are available.

The Boolean satisfiability problem (SAT) asks whether there exists an assignment to the Boolean variables of a given propositional logic formula such that the formula evaluates to TRUE. This problem was the first that was proven to be NP-complete^5,6^. NP-complete problems are both NP-hard and in NP. Since no algorithms with polynomial runtime for NP-hard problems are known, solving NP-hard problems has long been considered to be intractable for real-world computers. Despite this result, coming from asymptotic analysis, modern SAT-solvers perform very well on solving large SAT instances originating from industry and academics, with formulas that have up to a million clauses^7^. At the moment of writing there exists no good general method or metric to predict if a given SAT instance is hard to solve. For practical applications it therefore makes sense to assess the performance of the solvers on the investigated instances by careful benchmarking instead of doing asymptotic analysis.

The approach considered in this work reduces factoring, a problem with a subexponential algorithm, to an NP-hard problem and then running (classical or quantum) solvers that have exponential runtime in the worst-case. At the surface, this obviously does not sound like a promising idea, as the quantum SAT solver must make up the exponential ground lost by translating the problem with subexponential algorithms to one where the best known algorithms are exponential. One might hope that good SAT solving heuristics for solving SAT on random or average-case instances could nevertheless have a practical impact on integer factorization.

The original goal of this project was to encode the RSA factoring challenges^8^ to SAT instances and see how well modern SAT solvers would perform on those instances. The smallest semi-prime of these challenges is RSA-100: a 100-digit or 330-bit number. This number was factored in a few days almost immediately after the challenge was posted^9^ in 1991, whereas the current record for factoring stands at factoring RSA-250: an 829-bit semi-prime^10^. The intention was to compare current state-of-the-art SAT solvers against the numerical results from 1991, but it turns out that even the smallest RSA semi-prime poses too big of a challenge for these solvers.

A more promising approach is to try to speed up the solution to some subroutine of the NFS, as is done in^11^. In particular, one could reduce some carefully chosen sub-problem solved within the number field sieve to SAT. The sub-problem should be chosen so that classically solving the SAT instance is roughly as costly as the usual approach to solving the sub-problem. In this case, any quantum speed-up for solving these SAT instances would lead to a faster implementation of the number field sieve. This approach is explored in^12^.

### Contributions

This work provides a numerical analysis on the hardness of factoring numbers by solving the corresponding satisfiability problem, thereby confirming the folklore that factoring numbers does indeed give “hard” SAT instances. This is done by measuring the speed of the currently fastest SAT solver. We justify the choice of numerical analysis over theoretical asymptotic analysis by applying some common analysis tools from modern SAT solving theory and the observation that the tools provide no good prediction for the actual runtime. We extrapolate the numerical results to investigate the asymptotic behavior of the solver and compare the results with the asymptotics of factoring with numerical algorithms. Finally, the results are used to estimate an upper bound on the speedup that can be achieved on this specific problem using currently known quantum algorithms.

As a minor contribution, we developed a tool that can create smaller SAT instances for factoring (using long multiplication) than any other publicly available tool. This tool and scripts for generating semi-primes and reproducing the results of this paper have been made available online^13^.

## SAT instances

An instance of the SAT problem is a formula in Boolean propositional logic. Every *variable* (*x*) can take the value TRUE or FALSE. An instance is said to be *satisfiable* if an assignment to the variables exists such that the overall formula evaluates to TRUE. Sometimes (as is the case when factoring via SAT) we are also interested in the *values* of the variables in the satisfying assignment itself. Formally this is no longer a decision problem, but we will sometimes be a bit informal in our language and discuss these as if they were decision problems.

This work considers CNF-SAT where all formulas are in conjunctive normal form (CNF): each formula must be a conjunction of disjunctions of literals. A *literal* is either a direct variable (*x*) or a negated variable (denoted $\bar{x}$), a disjunction of literals is called a *clause*. Further restricting each clause to exactly three literals would give the 3SAT problem. A satisfying assignment to CNF-SAT thus assigns a Boolean value to each variable such that at least one literal evaluates to TRUE in every clause. The CNF-SAT problem is equivalent to SAT^14^, in the sense that for each SAT instance an equisatisfiable CNF-SAT instance can easily be found with its size linear in the length of the original SAT instance. All tools we used for generating and solving SAT instances work with the DIMACS format which specifies formulas in CNF form.

A closely related problem is called CircuitSAT: given a Boolean circuit with a single output, is there an input such that the output is TRUE? One can translate any Boolean circuit into a Boolean formula: assign a variable to each input wire, then consider the logical operator corresponding to each gate (with a single output wire) in order. Each operator has a short CNF description, for example a NAND-gate (which forms a complete basis for Boolean formulas) with input wires *x*, *y* and output wire *z* has the corresponding formula $(x \vee z) \wedge (y \vee z) \wedge (\bar{x} \vee \bar{y} \vee \bar{z})$. Once the input wires are fixed to some value, there is only one possible value for the output wires such that the gate formula evaluates to TRUE. For example we fix can the input to $x=\text {TRUE}$, $y=\text {FALSE}$ by adding the clauses *x* and $\bar{y}$. A SAT-solver can examine those five clauses and find that the only satisfying assignment sets $z = \text{ TRUE }$. Combining gates to make a circuit is done by reusing output variables of earlier gates as input variables in later gates.

More interesting is to fix a value on the output variables of a circuit and ask the SAT-solver to find a satisfying assignment. For example adding the clause *z* to the NAND-gate gives three satisfying assignments: $x \wedge \bar{y}$, $\bar{x} \wedge y$, and $\bar{x} \wedge \bar{y}$. In general a circuit might have zero or more satisfying assignments. Effectively the SAT-solver is finding preimages to the function described by the circuit. An immediate cryptanalytic application that springs to mind is finding preimages to secure hash functions: indeed this has been done with varying results^15–17^. More general cryptanalytic applications can be found throughout literature^18^ and occur in modern benchmarks^7^, although asymmetrical cryptographic primitives are rarely targeted.

This work examines circuits that encode the multiplication of two integers *p* and *q*. We fix the multiplication output bits of the circuit to the bit-values of the semi-prime *N* and ask the SAT-solver to find a satisfying assignment. Only two exist (trivial solutions are excluded by the problem encoding) those representing $N=pq$ and $N=qp$, so from the assignment one can read the factorization of *N*. For the remainder of this paper *n* represents the size of *N* in bits. We limit *p* and *q* similar to how the RSA cryptosystem limits its parameters: both need to be equally sized primes. We interpreted this last requirement to mean that their most significant bit may differ by at most one position.

### Encoding

Despite the asymptotic worst-case exponential runtime associated with SAT instances, it is not trivial to generate “hard” SAT instances: instances where the solver runtime grows exponentially in the number of variables. For several problems (encoded as a SAT problem) it turns out that modern SAT solvers can solve many instances in short time in practice. Specialized tools such as ToughSat^19^ exist that can generate SAT instances that are hard on average, based on problems such as integer factorization.

Multiplying larger integers requires larger circuits, which leads to instances with more variables and clauses, which leads to longer solving times. However, there are many choices to make when computing multiplication in a circuit and each choice will lead to different encodings of the SAT instance and a different solver runtime. For SAT solvers in general it turns out that the details of the encoding of a problem (beyond metrics such as number of variables and clauses) can have a significant impact on the solver runtime. The first choice is to consider different multiplication algorithms: a simple one and a more complex encoding that in theory leads to smaller instances.

Long multiplication (or schoolbook multiplication) is computed by multiplying *p* by each digit (bit) of *q* and adding the shifted results. For multiplying two *m*-bit numbers (where $m=n/2$) this requires $\Theta (m^2)$ bitwise multiplications and additions. The exact number of operations depends mainly on the circuit used for addition: our tool for generating instances^13^ minimizes the number of both variables and clauses by maximizing the number of full-adders used in the circuit. Counting the variables in the generated instances and applying regression reveals that the number of variables grows approximately as $0.750n^2 + 0.496n - 2.05$ and similarly the number of clauses grows as $4.25n^2 - 4.01n - 9.87$ with on average 3.31 literals per clause.

Karatsuba multiplication^20^ asymptotically improves upon long multiplication by a divide-and-conquer strategy and requires only $\Theta (m^{\log _2 3})$ multiplications at the cost of requiring more additions. The instances we tested were generated by the ToughSat application^19^ and contain approximately $2.59n^{\log _2 3} - 7.57n + 8.75$ variables and $61.5n^{\log _2 3} - 170n - 386$ clauses with on average 6.77 literals per clause. Inspection of the generated instances reveals that the Karatsuba circuits were built from more complex gates, which explains why there are more literals per clause. It is likely that building the Karatsuba circuit with a similar gate set would increase the number of variables and clauses by another (constant) factor.

Asymptotically the Karatsuba algorithm is not the best known algorithm and is outperformed by for example Toom-Cook or FFT-multiplication. These methods introduce additional overhead that is especially significant for small instances, where it would result in larger SAT instances. Given that we also observed only a minor difference in the runtime of long multiplication and Karatsuba instances, we decided not to encode these more complex multiplication algorithms.

Hardware design provides alternative multiplication algorithms, which are often optimized to minimize latency and for various other physical constraints. There is no indication that these optimizations are related to optimizations that lead to smaller and/or easier SAT instances. In fact our adder encoded in the SAT instances minimizes the number of half-adders required, which gives the smallest number of variables and clauses and results in the fastest SAT solver times, but the resulting clauses encode a circuit that would give extremely high latency if built from physical components.

Since the multiplication circuit is the same for each semi-prime of the same bitlength there is an alternative strategy we can apply when we want to factor only one semi-prime out of a polynomial sized set. We encode the multiplication circuit once and then “fanout” the resulting wires to one circuit per semi-prime that checks if the output equals that semi-prime. Those results are combined with a large OR-gate, so that the entire instance evaluates to TRUE if the multiplication outcome is equal to any of the semi-primes. By inspecting which values were assigned on the circuit input wires by the solver we learn which of the semi-primes it actually factored. The idea behind this encoding is that if there is an easy semi-prime somewhere in the input, then the solver itself may detect this and focus on solving that instance. As long as we encode only polynomially many semi-primes in the instance, the total instance size will remain polynomial.

An alternative solution for factoring numbers with SAT is to encode the integer division circuit $N / p = q + r$ and fixing the input value *N* and output remainder $r=0$. The rationale for this encoding is that the solver would only have to assign values to the bits of *p* and can then deterministically evaluate the entire circuit and check if the remainder is zero. However, in practice this encoding leads to substantially larger SAT-instances and tests with various solvers indicate that solving such instances is significantly slower, so we did not investigate this encoding any further.

A more promising approach is to reduce some subroutine of the number field sieve (NFS) to SAT where there is little or no increase in complexity by mapping to SAT, analogous to the approach taken by Bernstein, Biasse and Mosca^11^. In this case, even a small quantum speed-up will lead to a faster integer factorization algorithm. This approach is studied in detail in^12^.

## Classical solvers

Modern SAT solvers come in two classes. Conflict-Driven Clause Learning (CDCL)^21,22^ combines conflict analysis with branch heuristics to systematically backtrack the search-space of an instance. Stochastic local search approaches such as employed by WalkSAT^23^ or simulated annealing combine randomized assignments with probabilistic updates to find assignments that minimize the number of clauses violated. We found that for the semi-prime instances CDCL solvers outperformed the local search solvers by an order of magnitude. The scope of this project is limited to the black-box analysis of publicly available SAT solvers. This means we will not investigate the internals of the solvers for analysis of the runtime, nor do we allow domain-specific knowledge to speed up solver times.

When considering the runtime *T* of an algorithm (either classical or quantum) we are most interested in the runtime as a function of the input size. In order to determine if one solver is faster than the other, we should always consider the *total* runtime. We measure the total runtime of the SAT solver *including* the runtime of the preprocessor. Technically the measurement should also include the time for generating the SAT instances, but this is negligible compared to the solver time. For many classical solvers the total runtime can be naturally partitioned into the time spent in pre-/post-processing ($T_p$) and the time spent solving ($T_s$): $T(n) = T_p(n) + T_s(n)$ where *n* is the input size of the problem. Examples of this partitioning occur with the SAT preprocessor ($T_p$) and the SAT solver ($T_s$), the compiling ($T_p$) and running ($T_s$) of Shor’s algorithm or the creation of a Hamiltonian ($T_p$) and the execution of the adiabatic algorithm ($T_s$).

In order to properly analyze the runtime of any algorithm we need to consider *T*(*n*) and not just $T_s(n)$, since an unbounded amount of preprocessing can find a solution and render $T_s(n)$ to be trivial. We should also take care to set *n* to be the input size of the problem. Concretely this means we should let *n* be the size of the semi-prime and not the number of variables or clauses in our SAT instance. It is also important to analyse instance sizes larger than some lower bound ($n \ge n_0$), as the asymptotic behaviour is not visible for smaller sizes. For example the asymptotics of the MapleCOMSPS solver (discussed next) on integer factorization only become apparent at $n_0 = 20$ bit semi-primes.

We tested the MapleCOMSPS^24^ SAT solver for the simple reason that at the time of running the benchmarks this was the fastest solver according to the SAT Competition 2016^25^. We compiled and ran the solver with default settings, except for the random seed which was fixed for each call to the solver to ensure reproducibility of the results.

Another solver that we tested is CryptoMiniSat 5^26^, because it has “Automatic detection of cryptographic [...] instances”^27^. One might consider this to be cheating by using domain-specific knowledge and therefore it should not be included in the benchmarks. CryptoMiniSat appears to focus on symmetric cryptography and appears to provide no speedup on public cryptography instances, which we confirmed during an initial round of benchmarking. We inspected the (partial) results and found that CryptoMiniSat 5 was consistently being outperformed by MapleCOMSPS. For this reason we did not further analyze this solver, but the results can be found in the Supplementary Material.

All measurements were performed on a ThinkPad laptop with a 64-bit Intel Core i5–4200M (Haswell) CPU running at 2.50 GHz. All measurements were executed sequentially and on a single core. Where applicable we use regression to fit a line to the data and the goodness-of-fit is quantified by the $r^2$ parameter.

### Results

**Figure 1:**
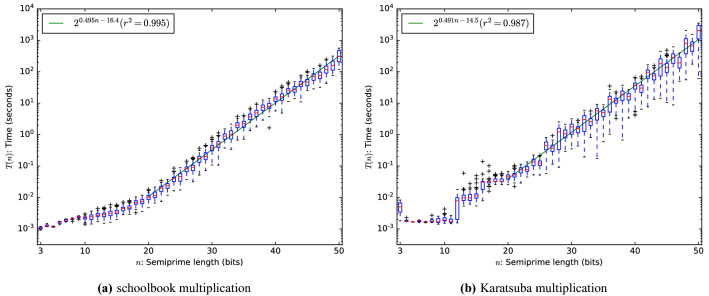
Runtime of MapleCOMSPS on factoring semi-primes.

Usually when analyzing the runtime of a randomized algorithm we are interested in the expected runtime: the mean computed over the random bits. We do this by factoring the same number multiple times using a different PRNG-seed for the solver and average the runtime to compute the expected runtime numerically. We are interested in the asymptotics: the growth of the runtime as a function of the size of its input, so we group the semi-primes by their bitlength *n* (100 semi-primes per bitlength) and plot the mean runtime of solving five times. The results are given in Fig. 1a and are showing an exponential trend. The green line is fitted against the median runtime of all semi-primes of the same bitlength.

We repeated the same experiment for multiplication with the Karatsuba algorithm. The results are given in Fig. 1b: note that asymptotic runtime has improved somewhat over schoolbook multiplication at the cost of a larger constant. We conclude that changing the multiplication algorithm does not make factoring with SAT solvers efficient. Since the larger constant dominates the runtime at this small scale, we will consider schoolbook multiplication for the remainder of our experiments.

**Figure 2:**
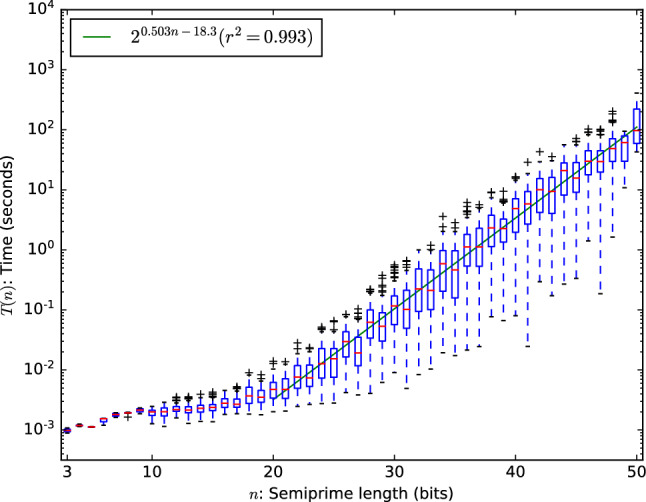
Minimum runtime of MapleCOMSPS on factoring semi-primes using schoolbook multiplication.

An alternative strategy for factoring is to run several solvers in parallel and wait for the first one to return a solution. We simulate this strategy by taking the minimum solver time of solving the same instance with the solver initialized with 100 different random seeds for 100 semi-primes per bitlength: the results are given in Fig. 2. Asymptotically the runtime became worse by employing this strategy. Note that this strategy does push down the constant by approximately $2^{6.1}$. Since this is smaller than 100 it does not lead to a lower expected runtime on this small scale when we consider the total runtime of all parallel solvers.

We can also see in Fig. 2 that some semi-primes are significantly easier to solve than others with this strategy. Even if we only manage to factor some semi-primes that may be important to (for example) cryptography. For this method to be asymptotically efficient, it is required that the runtime is pushed down exponentially for more than just negligibly many cases. To see if it does we can inspect the distribution of the solver runtime given different seeds. Here we focus on three different semi-primes: the easiest, average and hardest semi-prime from the 100 semi-primes of 35 bits, where hardness is defined by the expected (mean) solve time computed over 360 seeds. The distribution for all other semi-primes can be generated at^13^.

**Figure 3:**
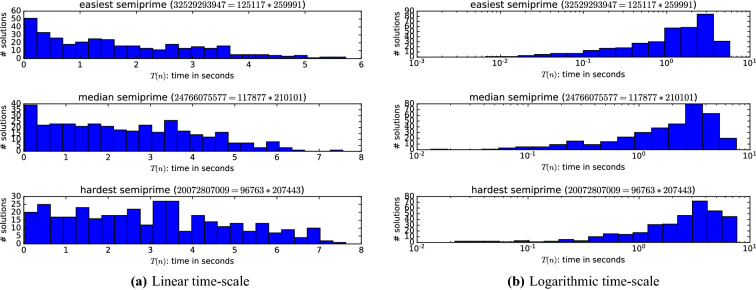
Histogram of the MapleCOMSPS runtime on factoring semi-primes using schoolbook multiplication.

Although no strong conclusions should be drawn from the results in Fig. 3a, the distribution does suggest that running a few parallel solvers may lower the total runtime. To see if it may be considered efficient we again inspect the distribution but this time on a logarithmic scale: see Fig. 3b.

This data suggests that even if the method could push down the runtime significantly for any semi-prime, it only does so with negligible probability. Another way of interpreting this data is that employing parallel SAT solvers to factor a semi-prime does not appear to be better than employing a single solver.

**Figure 4:**
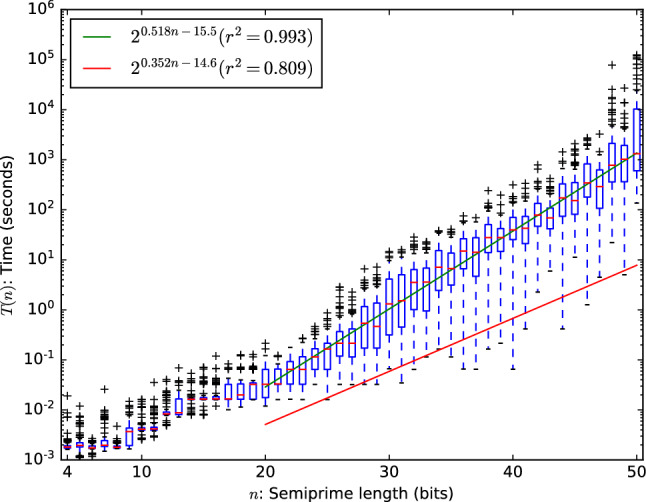
Runtime of MapleCOMSPS on factoring one of 100 semi-primes encoded in each instance using schoolbook multiplication.

The last strategy we investigate is that of encoding multiple semi-primes into a single instance: for example an adversary may interested into breaking just one out of many cryptographic keys. We encoded 100 semi-primes per bitlength in each instance and solved it 100 times using different seeds. The results are given in Fig. 4. Note that whereas the vertical boxplots in previous plots show a distribution over different primes, here a distribution over different solver PRNG-seeds is shown. From the data we conclude that this strategy is less efficient than solving instances with a single semi-prime. From inspection of the solver solution we can see which semi-prime was factored (see^13^). This reveals that some semi-primes in the same instance are factored more often than others, suggesting that these are easier to factor by the solver, although we note that these are not “easy enough” to make the overall method efficient. The supplementary information contains further analysis of patterns in the SAT instances, but finds no pattern that can be exploited for significantly faster solver times.

### Comparison to number-theoretical methods

**Figure 5:**
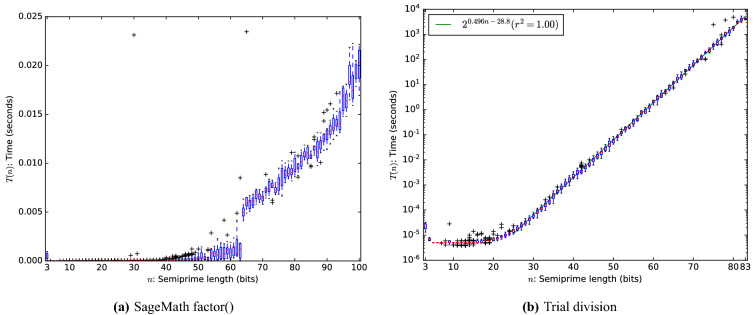
Runtime of factoring using numerical methods. No randomization was applied for obtaining these results.

One can put the above results in context by comparing the absolute runtime to that of other number-theoretical results. Using SageMath^28^ we measured the runtime of two approaches: factoring with the built-in factor function (Fig. 5a) and factoring by trial division (Fig. 5b).

SageMath is able to factor almost all semi-primes up to a 100 bits in under 0.025 s. The tested semi-primes are so small that the asymptotic behavior of the underlying algorithm is not even visible yet, so there is no point in extrapolating these results. In fact the cross-over point where the NFS is faster than asymptotically slower methods such as the quadratic sieve and the elliptic-curve method is much larger than 100 bits, so that SageMath is not even using NFS to factor these small numbers. Instead, we refer to the literature to find that the best classical factoring algorithm (the general number field sieve^29^) runs in $\exp \big ( ((64/9)^{1/3} + o(1)) (\ln N)^{1/3} (\ln N)^{2/3} \big ) = L_N[2/3, {(64/9)}^{1/3}]$ and this was indeed used to factor a 829-bit RSA modulus in approximately 2700 core-years^10^.

The timing of factoring using trial division is shown in Fig. 5b. The results reveal an exponential trend and with a much smaller constant than the SAT solver. On this small scale on which measurements were performed, trial division easily outperforms the SAT solvers. The asymptotic runtime of the methods are so close together that we cannot meaningfully extrapolate the results to find a cross-over point where the SAT solvers become faster than trial division. We therefore cannot rule out that factoring with classical SAT solvers is always slower than trial division.

## Quantum solvers

State of the art classical factoring algorithms have super-polynomial expected runtime $L_N[1/3, (64/9)^{1/3}]$^29^, whereas Shor’s algorithm^30^ runs in expected polynomial time. This algorithm requires a fault-tolerant quantum computer and no scalable version has been implemented yet. Shor’s algorithm has profound practical implications for currently deployed public-key cryptography such as RSA and the timing of the factoring of 1024-bit, 2048-bit or even larger semi-primes is of great practical significance for both contemporary and future security systems^31^. Mitigations for future systems and current systems requiring long-term security are being researched by the field of post-quantum cryptography^32–34^.

An interesting notion of quantum computing has been proposed by Farhi et al.^35^ in the form of adiabatic quantum computers. It was suggested that adiabatic quantum algorithms may be able to outperform classical computers on hard instances of NP-complete problems^36^. Since then, adiabatic quantum computation (a generalization of the adiabatic optimization explored deeply by Farhi et al.) has been proven to be polynomially equivalent to quantum computation in the standard gate model^37^. While the possibility of super-polynomial (or even just super-quadratic) quantum speed-up for NP-hard problems remains an open question it is generally believed that quantum computers (including adiabatic quantum computers) are not able to efficiently solve NP-hard problems such as SAT. It is known that any such speed-up must go beyond pure “black-box” search^38^ as attempted by Farhi et al.^36^ and must somehow exploit additional knowledge or structure^39^. Note that this assumption is implicit, e.g. in the fact that post-quantum cryptographers are working on the assumption that symmetric algorithms like AES and SHA that offers *n* bits of security against classical attacks offer *n*/2 bits of security against the best known quantum attacks^33^ (excluding some specific attacks in the “quantum superposition” attack model^40^). In this section we consider the speedup that can be achieved by reducing the problem of factoring a semi-prime to an instance of an NP-hard problem which is then solved with a quantum computer.

### Faster SAT solvers

One might hope that we can apply a quantum strategy that can improve on the best known classical methods. We chose SAT solvers to represent the best classical methods as their implementations are the highly optimized result of years of research. Generic quantum searching methods can achieve at most a quadratic speed-up, and we are aware of no convincing evidence that more than a quadratic speed-up can be achieved by quantum SAT-solving methods. For example, many modern SAT solvers rely on machine-learning techniques^24^ and many quantum methods with a quadratic speedup are known for a variety of machine-learning algorithms^41^. See also^42^ for why the exponential speedup promised in some quantum machine-learning literature is unlikely to be achieved in real-world implementations.

A quick calculation shows that even with a quadratic speedup, this strategy is not a very efficient one. We set an upper bound on the number of operations required for the classical solver based on our results. Accounting for any internal parallelism in the processor (four arithmetic ports per processor) and assuming that the CPU was fully occupied at every clock cycle this means that $10^{10}$ operations were being executed every second during the solving time.

**Figure 6:**
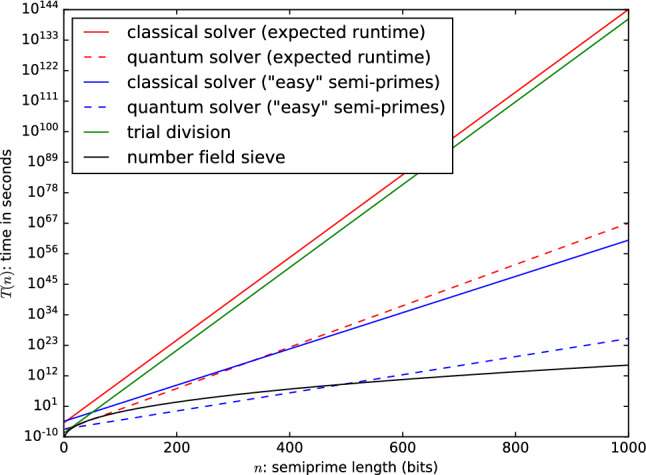
Comparison of efficiency of various factoring methods. The classical results are extrapolated from experimental data. The quantum results apply a quadratic speedup over the full classical computation. The number field sieve result plots $L_N[1/3, (64/9)^{1/3}]$ operations assuming the same number of operations per second.

Under this assumption the expected number of operations required for classical SAT solving becomes ${2^{16.8}\times 2^{0.495n}}$. With a quantum computer we might hope to reduce this to $\sqrt{2^{16.8}\times 2^{0.495n}} = {2^{8.41}\times 2^{0.247n}}$ operations. To put this in perspective, consider a quantum computer that can execute $10^{40}$ *quantum* operations per second. Note that even a classical computer with such speeds could break AES-128, SHA-256, RSA-2048 and ECC P-256 in an instant, so this is a very generous upper bound. Under these assumptions it would still take approximately a hundred times the lifetime of the universe to factor the RSA-250 number using the quantum SAT solving approach, whereas this number has been factored classically on a real machine in roughly 2700 core-years using number-theoretical methods. A visual comparison of these results are given in Fig. 6.

Note that all estimates so far are biased towards more (classical) operations per second. The reason is that we want to compute an upper bound on the speedup that can be achieved by applying Grover’s algorithm (or some alternative quadratic speedup) in order to factor numbers with SAT solving. It is almost certain that the processor executed less operations and it is very unlikely that the quadratic speedup can be applied to the full computation without any overhead of executing the algorithm. Therefore, classical algorithms will likely require less operations than reported and quantum algorithms will likely require more operations than computed.

Note also that the known speedups for quantum solvers are applied to $T_s$, even though the above calculation generously assumes that $T_p(n) = 0$ and the speedup can be applied to the full calculation time *T*(*n*). For most classical solvers it indeed holds that $T_p(n) \ll T_s(n)$ as *n* grows large enough, but for many of the adiabatic factoring methods discussed below it holds that $T_p(n) \gg T_s(n)$ as *n* grows. This means that our calculation is a significant overestimation of the maximum speedup that can be achieved with the adiabatic factoring method.

### Adiabatic factoring

The method used for factoring with the adiabatic algorithm first reduces factorization to finding the roots in a set of integer equations in which the unknown variables are restricted to binary values, corresponding to the input bits of the prime and carry bits of the intermediate computation. This is translated to the pseudo-Boolean optimization problem by squaring all equations (so that the roots correspond to the minimum values) and summing over all equations, resulting in a quartic polynomial. This reduction was first suggested by Burges as a method for generating unconstrained optimization problems whose complexity can be easily controlled^43^. The adiabatic algorithm^35^ is particularly well suited for encoding optimization problems of this kind: the resulting sum describes a Hamiltonian whose ground state encodes the solution and every variable corresponds to a single qubit. In general it is not easy to physically initialize the system in the ground state of the problem Hamiltonian. Instead the adiabatic method intializes the system in the ground state of an easier Hamiltonian. The adiabatic theorem tells us that if we evolve the physical system from the initial Hamiltonian to the final Hamiltonian slow enough, the system will remain in its ground state. Measuring the final state will then provide the answer to the optimization problem.

To assess the power of the adiabatic algorithm it is therefore important to quantify how fast this evolution can be done. A coarse lower bound is given by the spectral width of the time-dependent Hamiltonian, but sharper bounds on the runtime so far elude us^39^. This has led some researchers to study the applicability of the adiabatic algorithm to some NP-complete problems^36^. Most evidence for a speed-up is based on noise-free simulations on small instances (for which the asymptotic behaviour might not be visible) which are chosen randomly, shedding light on typical performance for small instances. Cryptographic problems require average-case hardness in order to be practical, which is why they are so suitable for testing worst-case behaviour of algorithms that solve them, especially when the reduction to an NP-hard problem is as simple as reducing factoring to SAT as demonstrated in the previous section.

Pseudo-Boolean optimization is known to be NP-hard, meaning amongst other things that a polynomial reduction exists from the SAT problem^44^. Real-world demonstrations of the adiabatic algorithm suffer from additional limitations (besides noise-resistance) in the number of available qubits and multi-qubit interactions. The latter limitation means that quartic terms in the objective function cannot always be realized. Using quadratization^45^ each objective function can be simplified to a quadratic polynomial at the price of additional variables, giving an instance of the well-studied quadratic unconstrained binary optimization (QUBO) problem. This simplification runs in polynomial time and results in only polynomially many variables overhead, so the problems are equivalent.

However for many real-world systems the extra variables (qubits) are not available, so additional simplifications are required. This is fine as long as these simplification steps do not dominate the overall runtime of the program. More precisely we can execute polynomially many simplification operations and $T_p(n)$ will remain polynomial in *n*, thereby not significantly increasing the runtime *T*(*n*) which is dominated by the super-polynomial runtime $T_s(n)$. When the simplification process is allowed to have an exponential runtime it can absorb the hardness of the problem, leaving a weaker problem to be solved (trivially) in polynomial time.

#### Implementations

The first adiabatic factorization^46^ was implemented in 2008 using nuclear magnetic resonance (NMR) to factor 21 using three qubits. The authors fit a quadratic curve against a theoretical approximation in a noiseless model, they measure the runtime as a function of the number of input qubits (not the size of the factored number) and they only consider the small domain of seven to sixteen input qubits. The same method has factored 551 by applying some preprocessing first^47^. It is doubtful that such small instances are a good indicator of polynomial asymptotic behaviour.

Later work^48^ translates the problem of factoring 143 into a pseudo-binary optimization instance, which is an NP-hard problem^44^. The authors manage this by introducing the additional assumption that both factors must be of equal bitlength with the most significant bit set to one. Combining these assumption with some simplifications in the pseudo-Boolean equations simplifies the problem so that it only concerns four input bits of the prime factors. Although the used simplifications are efficient, only an upper bound of their effectiveness is given.

Subsequent research^49^ observes that a minor generalization of the previous method reduces the problem to four input qubits whenever the two primes composing the semi-prime differ only in two positions, which likely occurs for infinitely many semi-primes^50^. This provides some evidence that the simplifications do not generalize and the factored number 143 was identified as a particularly easy number to factor. In other words, this example was hand-picked from an exponentially unlikely family of semi-primes that are by design easy to factor. The authors report the number 56,153 as being the largest semi-prime factored quantumly and at the same time argue that the work has factored an arbitrarily large set of semi-primes (since they can be pre-processed into solving the same pseudo-Boolean equations). The reason for not reporting a bigger number appears to be the large runtime $T_p$ of the simplification process.

Much subsequent research in the adiabatic factoring field has focussed on methods such as deduc–reduc^51^, split-reduc^52^ and energy landscape manipulation^53^, all of which can be seen as improvements on the preprocessor runtime $T_p$ and none of which do any improvements on $T_s$.

The problems with viewing these works as relevant quantum integer factorization benchmarks is highlighted even further in the more recent paper that claims to have factored 291,311 with adiabatic quantum computation^54^. The authors take the above approach and reduce the problem of factoring 291,311 to the integer equations $q_1 + q_2 - 2 q_1 q_2 = 1$, $q_2 + q_5 - 2 q_2 q_5 = 0$, and $q_1 + q_5 - 2 q_1 q_5 = 1$. where the variables $q_i$ must take on binary values and represent unknown bits in the binary representation of factor $q = 1000 q_5 01 q_2 q_1 1$. The authors stop their simplification process at this point and fail to notice that the above equations can be further simplified to $q_1 = 1 - q_2 = 1 - q_5$. Both solutions $q_1 = 0$ and $q_1 = 1$ correspond with respective factors $q = 557$ and $q = 523$. In other words, the number was already factored by the simplification process and the adiabatic quantum computation was a complicated way of flipping a coin and deciding between the two factors. The above criticism of these claims to meaningful quantum factoring benchmarks was in fact already made in 2013^55^.

A method called Variational Quantum Factoring (VQF)^56^ employs the same strategy for factoring, which is to reduce it to an NP-hard optimization problem. The authors are careful to ensure that preprocessing takes only polynomial time. Although the authors claim that “VQF could be competitive with Shor’s algorithm even in the regime of fault-tolerant quantum computation”, we find no convincing argument to support this conjecture. In particular, they do not provide convincing evidence that the solving step is efficient: no semi-prime larger than $2^{15}$ is considered by their work and they observe that “the mere presence of carry bits negatively affects the algorithm”.

The criticism from^55^ applies equally well against “compiled versions” of Shor’s algorithm (where partial knowledge of the factors is used to specify the quantum circuit): both implementations require much precomputation and therefore do not scale to factoring larger numbers. The problem is that both precomputations require prior knowledge of the solution. “Compiled versions” of Shor’s algorithm were never intended to scale to meaningful input sizes, as is highlighted in the abstract of the work factoring 15 with NMR: “scalability is not implied by the present work. The significance of our work lies in the demonstration of experimental and theoretical techniques”^57^.

The important difference is that the runtime of Shor’s algorithm is well understood and provides a super-polynomial speedup in $T_s$ over even the best numerical methods for factoring. As fault-tolerant hardware emerges, we can simply strip away the non-scalable optimizations. On the other hand the runtime of reducing factoring to an NP-hard problem and then solving it with (quantum) solvers is not understood very well, but the evidence provided in this work points in the direction that it cannot even compete with classical numerical methods for factoring.

### D-Wave

The D-Wave systems work by a process called quantum annealing, which can be viewed as a noisy version of adiabatic quantum computing. It has been shown that $O(n^2)$ qubits suffice to encode factoring into a quantum annealing instance with local interactions^58^. The article “Boosting integer factoring performance via quantum annealing offsets”^59^ describes a “boost” when comparing factoring on the D-Wave machine with annealing offsets against the D-Wave machine without annealing offsets. The largest factored number has 20 bits.

All semi-primes up to 200,000 (18 bits) have been factored with help of the D-Wave 2X by heuristically mapping the optimization problem to the Chimera graph underlying the machine^60^. Exponential methods from computational algebraic geometry are used for preprocessing the instances without quantification of the (asymptotic or measured) runtime so that there is no indication of the efficiency of this preprocessing step. Although some statistics on the annealing process are provided for six semi-primes, not enough information is given for a meaningful assessment on the scalability of both the efficiency and effectiveness of this method.

Integer factorization has been implemented many times on the D-Wave 2000Q by similar strategies. While early experiments only factored four semi-primes^61^, later work^62,63^ has factored more numbers by reducing the required number of qubits through more preprocessing (in polylogarithmic time). Wang et al.^63^ claim $O(n^2)$ annealing runtime without any justification; this unjustified claim seems incorrect, especially when considering the observation by Peng et al.^62^ that the rapidly decreasing accuracy limits the scalability of the method. None of these works presents convincing evidence that quantum annealing will find factors with significant likelihood in polynomial (or even sub-exponential) time.

A similar method was developed independently by Kieu^64^ and Yan et al.^65^. Their method translates factoring into an (NP-hard) optimization problem of minimizing $(N - pq)^2$ (or a similar expression), by encoding that directly in the problem Hamiltonian. Besides problems in translating the work to the Boolean logic required by the D-Wave machine^66^, the method has an exponential cost in energy in order to be efficient in time.

## Discussion

SAT solvers are not known or believed to be able to factor semi-primes efficiently. Overall, even the fastest solver (MapleCOMSPS) has an exponential runtime in the size of the factors. Closer inspection of the solver runtime indicates that the solver is not able to detect any pattern in the SAT formulas that encode the factorization problem. Asymptotically the solver runtime appears to be comparable to that of trial division, but this advantage is almost completely negated by the overhead in the constant term. The performance of SAT solvers does not even come close to that of number-theoretical methods.

Of course if it were that easy RSA would be broken regularly by SAT solvers which is not the case. Furthermore, in practice it appears that SAT instances derived from integer factorization instances are hard SAT instances. Thus it would be especially surprising if a SAT solver could solve these instances with resources comparable to that of using the classical number field sieve (i.e. subexponential complexity).

Quantum SAT solvers are not expected to do much better. Published results from experiments on quantum hardware lack the details to conclude exactly how big of a quantum speedup can be practically achieved, but it certainly seems insufficient to make up for the gap introduced by switching from subexponential algorithms to (worst-case) exponential ones. Even when calculating a very optimistic quantum speedup to the current state-of-the-art classical solvers, these solvers are outperformed with orders of magnitude by (classical) number-theoretical factoring methods.

Our work explores the possibility of a quantum speedup more deeply and reinforces the folklore that reducing multiplication to SAT and then applying SAT solvers, classical or quantum, is not useful for factoring numbers of sizes relevant to cryptography.

Of course, one cannot rule out unexpected breakthroughs in quantum SAT solving or a wide range of other quantum or classical approaches to factoring semi-primes. However, it is important to distinguish the possibility of unexpected breakthroughs (especially those that contradict conventional wisdom or lack a plausible roadmap) from tracking progress of an existing hardware platform and of an algorithm that is pertinent for cryptographically relevant semi-primes (i.e. classical computers and the NFS). Once scalable fault-tolerant quantum computers capable of implementing Shor’s algorithm are available, a similar tracking would be very meaningful (with the caveat outlined in the introduction). In the meantime, it is important to track progress toward achieving scalable fault-tolerant quantum computers.

In other words, notwithstanding other scientific merits of these works, we are not aware of any evidence that any SAT-based quantum factoring results to date, including factorization by quantum annealing, are relevant milestones toward large-scale integer factorization or the demonstration of a speed-up over the best known classical algorithms for integer factorization.

## Supplementary Information

Supplementary Information.
