# Supplementary material for: Factoring semi-primes with (quantum) SAT-solvers
Source: Sci Rep. 2022 May 14;12:7982. doi: 10.1038/s41598-022-11687-7 (PMC9107490; doi:10.1038/s41598-022-11687-7)
Supplement: Supplementary file 1 — Supplementary Information. [file 41598_2022_11687_MOESM1_ESM.pdf]

Supplementary Information
to “Factoring semi-primes with (quantum) SAT-solvers”

Michele Mosca and Sebastian R. Verschoor*
srverschoor@uwaterloo.ca

CryptoMiniSat 5

Fig. 1 shows the performance of factoring semi-primes with CryptoMiniSat 5. This solver solved each semi-prime SAT instance once, so no averaging has been applied to the shown results. In particular, one might be tempted to conclude from the longer whiskers in the depicted results that the CryptoMiniSat solver is lucky more often. However, the MapleCOMSPS solver gives similar results when only considering one solution. Closer inspection of the data reveals that CryptoMiniSat 5 is is outperformed consistently by MapleCOMSPS.

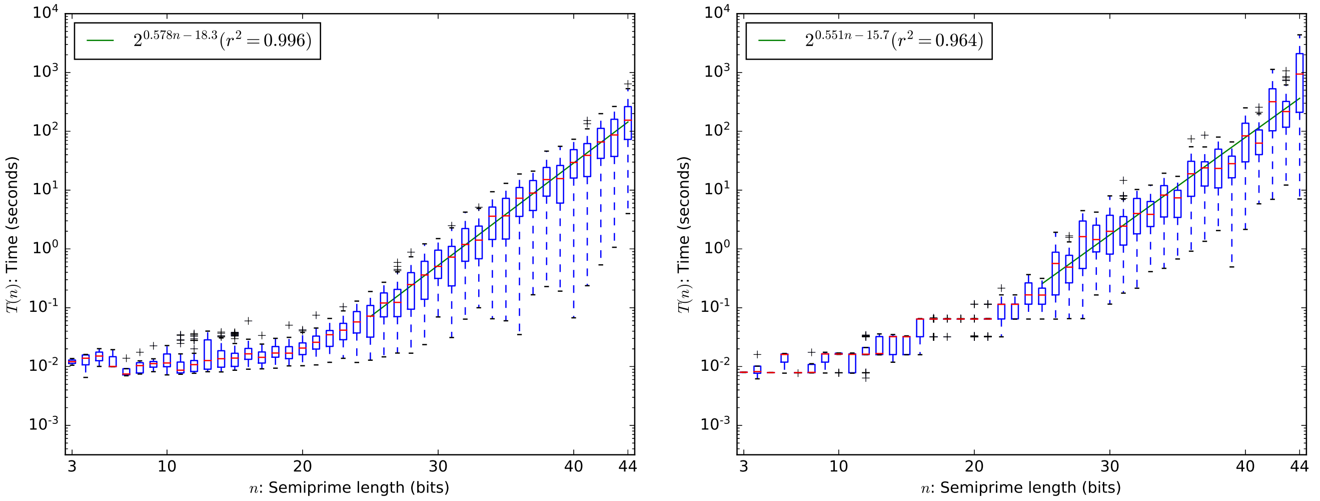

(a) schoolbook multiplication **(b)** Karatsuba multiplication

Figure 1. Runtime of CryptoMiniSat 5 on factoring semi-primes. We measured 100 semi-primes per bitlength and applied no randomization.

Patterns

The results in the main text support that it is hard *on average* to factor a number with SAT solvers, but we can also observe that some numbers are easier to factor than others. It is an interesting question whether there is some structure in the semi-primes that is picked up by solver that allows it to factor more efficiently or whether the solver’s heuristic choices accidentally lead to a faster solution. We try to answer this question by inspecting the instances using two analytic methods from the SAT literature (backdoors and community structure) and we do some manual inspection of the instances. Because we are interested in the fastest solver time, we focus on the minimum solver time per instance given different random seeds.

Backdoors

Backdoors in SAT instances were introduced by Williams, Gomes and Selman[1]. A backdoor is a subset of variables such that setting these variables to any value allows a so-called subsolver to assert if the entire formula is satisfiable in polynomial time. If the solver can find such a backdoor of size k with a subsolver that runs in time l, the entire solver can run in time $O(l2^k)$. Any CircuitSAT instance has a trivial backdoor in the form of the input wires/variables: set these and the rest of the clauses can be determined deterministically. (This is why we also encoded a division circuit: the input to that circuit contains one prime instead of two.) A backdoor subset for CircuitSAT therefore only becomes interesting when it is smaller than the set describing the input variables.

Every instance has n input wires, but the solver runtime suggests that a backdoor of $k \approx n/2$ variables was found. Given the structure of the problem this is not surprising (division to find the other k input bits only takes polynomial time), but it is

somewhat surprising given that it is unlikely that the SAT solver was programmed to perform this division. More meaningful analysis of this observation would require inspecting the internals of the solver to look for potential subsolvers and backdoor detection capabilities. We consider this outside the scope of this project. We simply conclude that even if a backdoor of size $n/2$ is found then the runtime of the SAT solver would still be exponential and therefore would not impact the security of RSA.

Community structure

A SAT instance can be represented as a graph where each variable is a vertex and an edge is drawn between vertices when the variables occur in the same graph. The community structure of a graph is often characterized by a quality metric Q (also known as the modularity of the graph). According to Newsham, Ganesh, Fischmeister, Audemard and Simon[2] the community structure of a SAT instance should provide us with a good prediction on how hard it is to solve that instance: instances are harder to solve when $0.05 \leq Q \leq 0.12$.

An immediate problem that occurs when applying the above theory to the generated circuits is that all instances for semi-primes of the same bitlength have the same structure: the encoded circuit is simply an m by m bit multiplier. Therefore, we compute the community structure only on the instances after they are simplified by the solver's preprocessor. We approximate the value of Q with the greedy algorithm by Clauset, Newman and Moore[3].

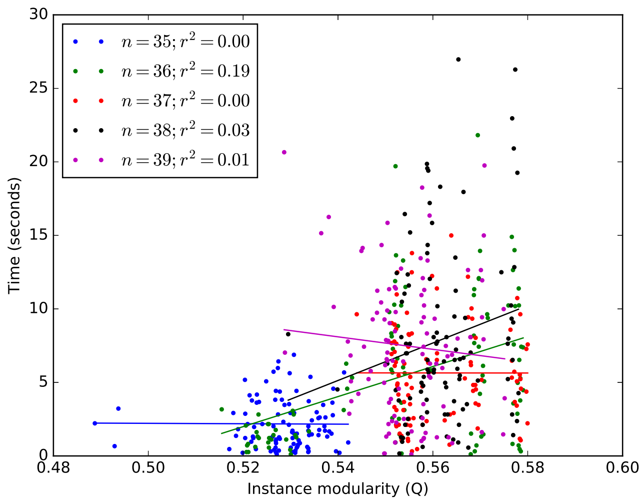

Figure 2. Solver times per community structure. We only display the results for some values of n to avoid more clutter, but similar results hold for all n.

Even after this preprocessing step the instances for long multiplication have too little variation to conclude anything about the relation between Q and solver time. For some Karatsuba instances the results are given in Fig. 2. The results are grouped according to the bitlength n and per group linear regression is applied to each group. The low r^2-values suggest there is no relation between modularity and solver time for these instances.

Interestingly, the values of Q are relatively high and far outside the range $0.05 \leq Q \leq 0.12$ for which the instances were conjectured to be hard, yet the instances are still hard to solve. The above data leads to the conclusion that the community structure does not provide a good prediction for solver runtime when applied to SAT instances that encode multiplication circuits.

Other metrics

Besides the above metrics that can be computed on any SAT instance, one might consider if there is any correlation between metrics that apply only to this specific use case. In particular we are interested if there is any pattern in N, p and/or q that the solver is able to exploit for a faster solving time. Since SAT instances are defined over Boolean variables we considered the Hamming weight of: N, p, q, and $p \oplus q$. We also measure if the solver is able to pick up on some patterns that make a number easier to factor according to number theoretic methods (such as Pollards $p-1$ method): smoothness of $p-1$, smoothness of $q-1$, $|p-q|$, and $\log N$. We measured the correlation with the solver time. No metric shows any significant correlation.

Fig. 3 and 4 examine the relation between various metrics on p, q and the solver time for long multiplication encoding and Karatsuba encoding (respectively). See also[4] for enlarged images. We examined bitwise patterns as these are most likely exploited by the SAT solver and we examined smoothness as this can determine the hardness of factoring for some number-theoretical methods.

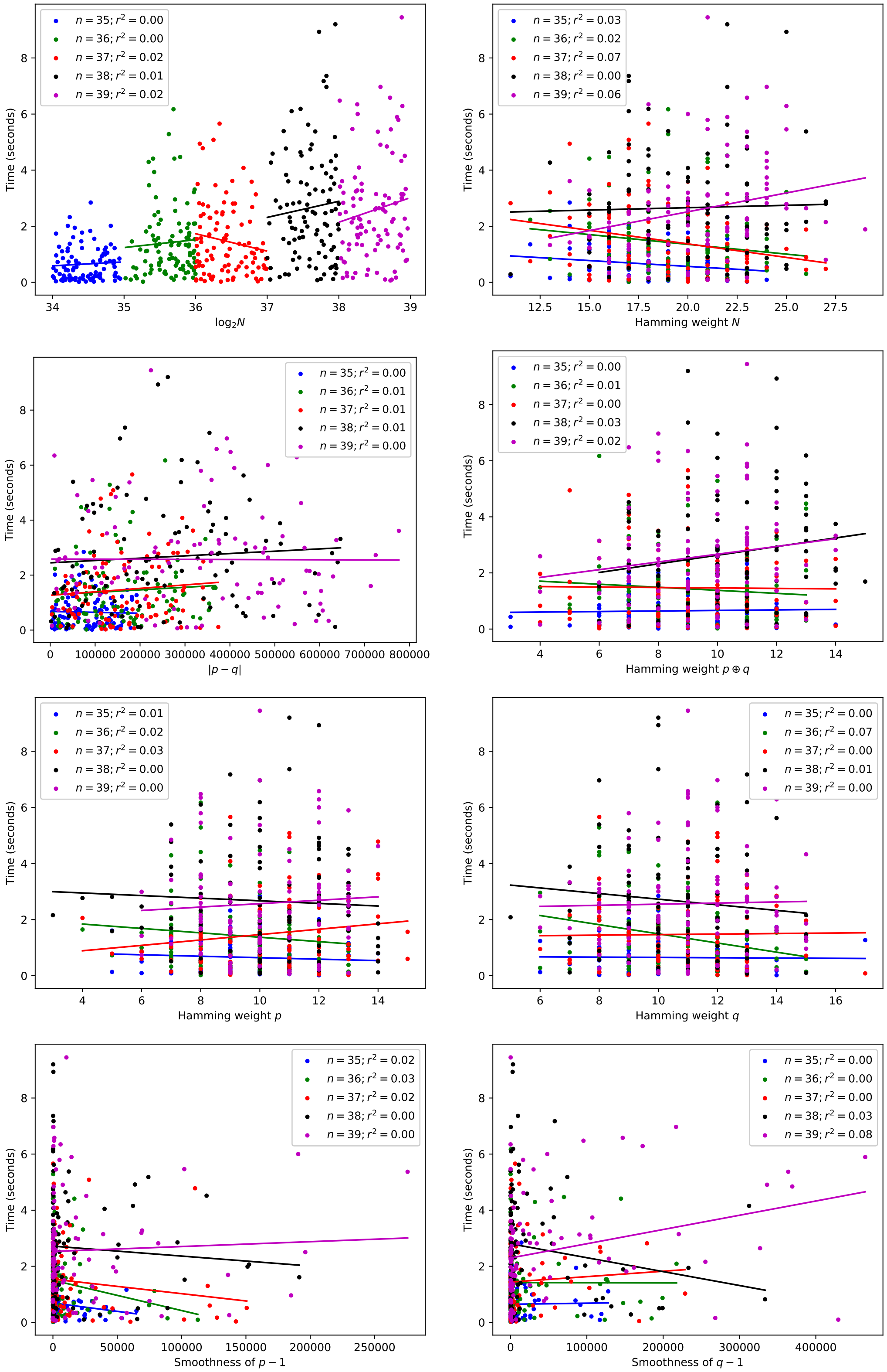

Figure 3. Solver time versus various patterns (schoolbook encoding).

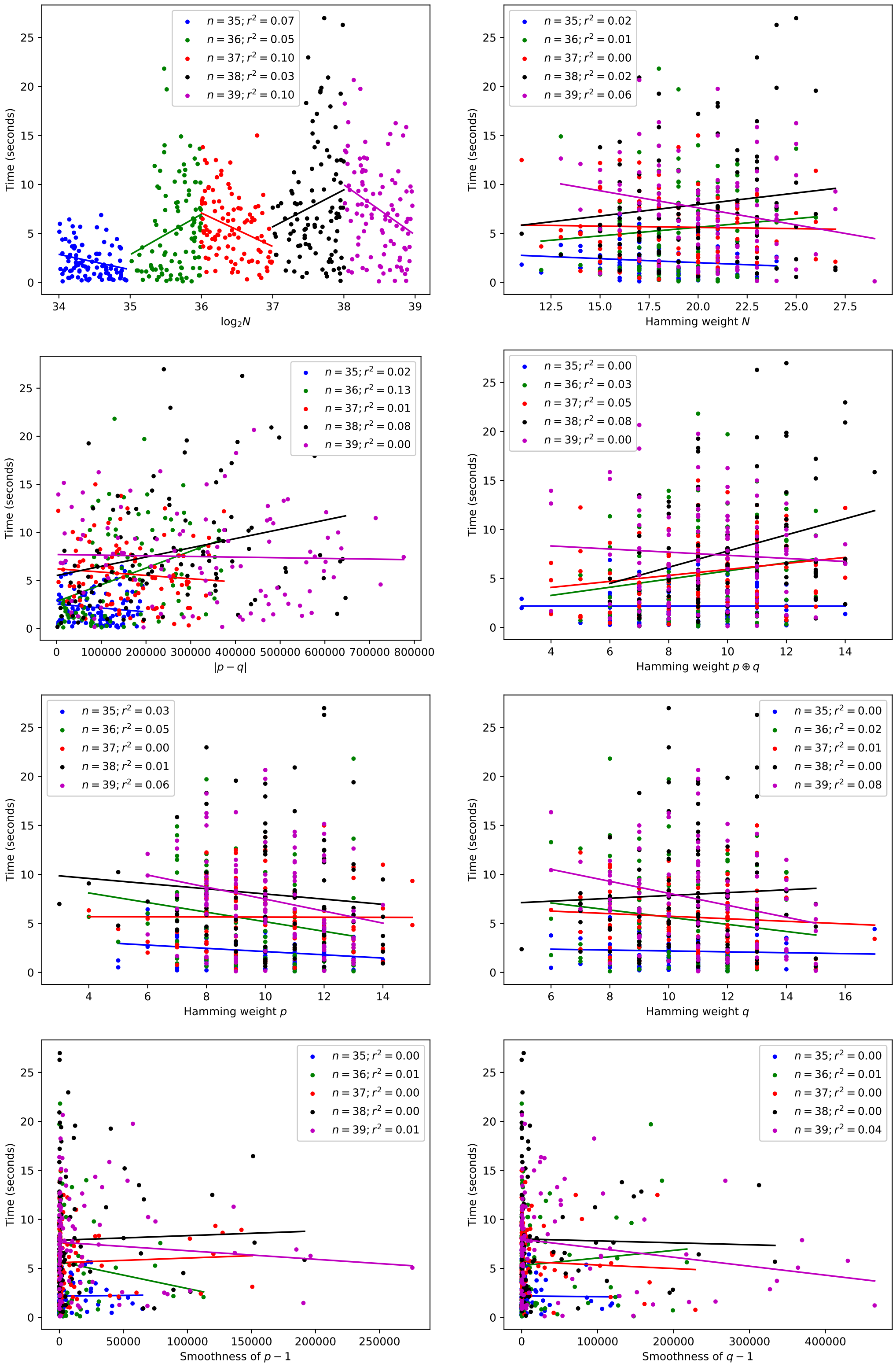

Figure 4. Solver time versus various patterns (Karatsuba encoding).

Note that only the first two metrics ($\log_2 N$ and Hamming weight(N)) could potentially be used to predict how fast the solver will find a solution. The remaining metrics require knowledge of the value of p and q, but these metrics could be important for anyone generating primes in the RSA cryptosystem.

However, the lack of any correlation indicates that none of the investigated patterns have a significant impact on the solver time. In other words, SAT solvers do not influence the method by which a user of the RSA cryptosystem should generate primes.

References

1. Williams, R., Gomes, C. P. & Selman, B. Backdoors to typical case complexity. In *IJCAI-03*, 1173–1178 (Acapulco, Mexico, 2003).

2. Newsham, Z., Ganesh, V., Fischmeister, S., Audemard, G. & Simon, L. Impact of community structure on SAT solver performance. In Sinz, C. & Egly, U. (eds.) *Theory and Applications of Satisfiability Testing - SAT 2014*, vol. 8561 of *Lecture Notes in Computer Science*, 252–268, DOI: https://doi.org/10.1007/978-3-319-09284-3_20 (Springer, Vienna, Austria, 2014).

3. Clauset, A., Newman, M. E. J. & Moore, C. Finding community structure in very large networks. *Phys. Rev. E* **70**, 066111, DOI: https://doi.org/10.1103/PhysRevE.70.066111 (2004).

4. Verschoor, S. R. SAT factoring. GitHub https://github.com/sebastianv89/factoring-sat (2019).
